# Virtual pterygoid implant planning in maxillary atrophic patients: prosthetic-driven planning and evaluation

**DOI:** 10.1186/s40729-023-00472-4

**Published:** 2023-03-27

**Authors:** Yuanyuan Sun, Chunfeng Xu, Ningtao Wang, Yiqun Wu, Yuelian Liu, Shengchi Fan, Feng Wang

**Affiliations:** 1 Department of Oral Cell Biology, Academic Center for Dentistry Amsterdam (ACTA), University of Amsterdam and Vrije Universiteit Amsterdam, Amsterdam, Netherlands; 2 Department of Second Dental Center, Shanghai Ninth People’s Hospital, Shanghai Jiao Tong University School of Medicine, College of Stomatology, Shanghai Jiao Tong University, National Center for Stomatology, National Clinical Research Center for Oral Diseases, Shanghai Key Laboratory of Stomatology, Shanghai Research Institute of Stomatology, No. 280, Mohe Road, Baoshan District, Shanghai, 201900 China; 3 Department of Oral and Maxillofacial Surgery, University Medical Center of the Johannes-Gutenberg University, Augustusplatz 2, 55131 Mainz, Germany

**Keywords:** Pterygoid implant, CBCT, Atrophic maxilla, Tilted implant

## Abstract

**Purpose:**

The study aims to use cone beam computed tomography (CBCT) to (1) define the virtual valid length of pterygoid implants in maxillary atrophic patients from the prosthetic prioritized driven position and (2) measure the implant length engaged in the pterygoid process according to the HU difference of the pterygoid maxillary junction.

**Materials and methods:**

Virtual pterygoid implants were planned with CBCT of maxillary atrophic patients in the software. The entry and angulation of the implant were planned according to the prosthetic prioritized driven position in the 3D reconstruction image. The planned implant length and the valid length defined as the implant between the pterygoid maxillary junction and pterygoid fossa were recorded. The relationship between the implant and sinus cavity was also evaluated.

**Results:**

A total of 120 CBCT samples were enrolled and virtually planned. The mean age of the patients was 56.2 ± 13.2 years. One hundred and sixteen samples could successfully place virtual implants according to the criterion. The mean implant length and mean implant length beyond the pterygoid maxillary junction were 16.3 ± 4.2 mm (range, 11.5–18 mm) and 7.1 ± 3.3 mm (range, 1.5–11.4 mm), respectively. Ninety percent of virtually planned implants had a close relationship with the sinus cavity, and implants exhibited longer lengths when they had no relation with the sinus.

**Conclusion:**

From a prosthetic prioritized driven position with fixed entry and angulation, pterygoid implants achieve adequate bone anchorage length beyond the pterygoid maxillary junction. Due to the individual anatomy and the volume of the maxillary sinus, the implants presented a different positional relationship with the maxillary sinus.

**Graphical Abstract:**

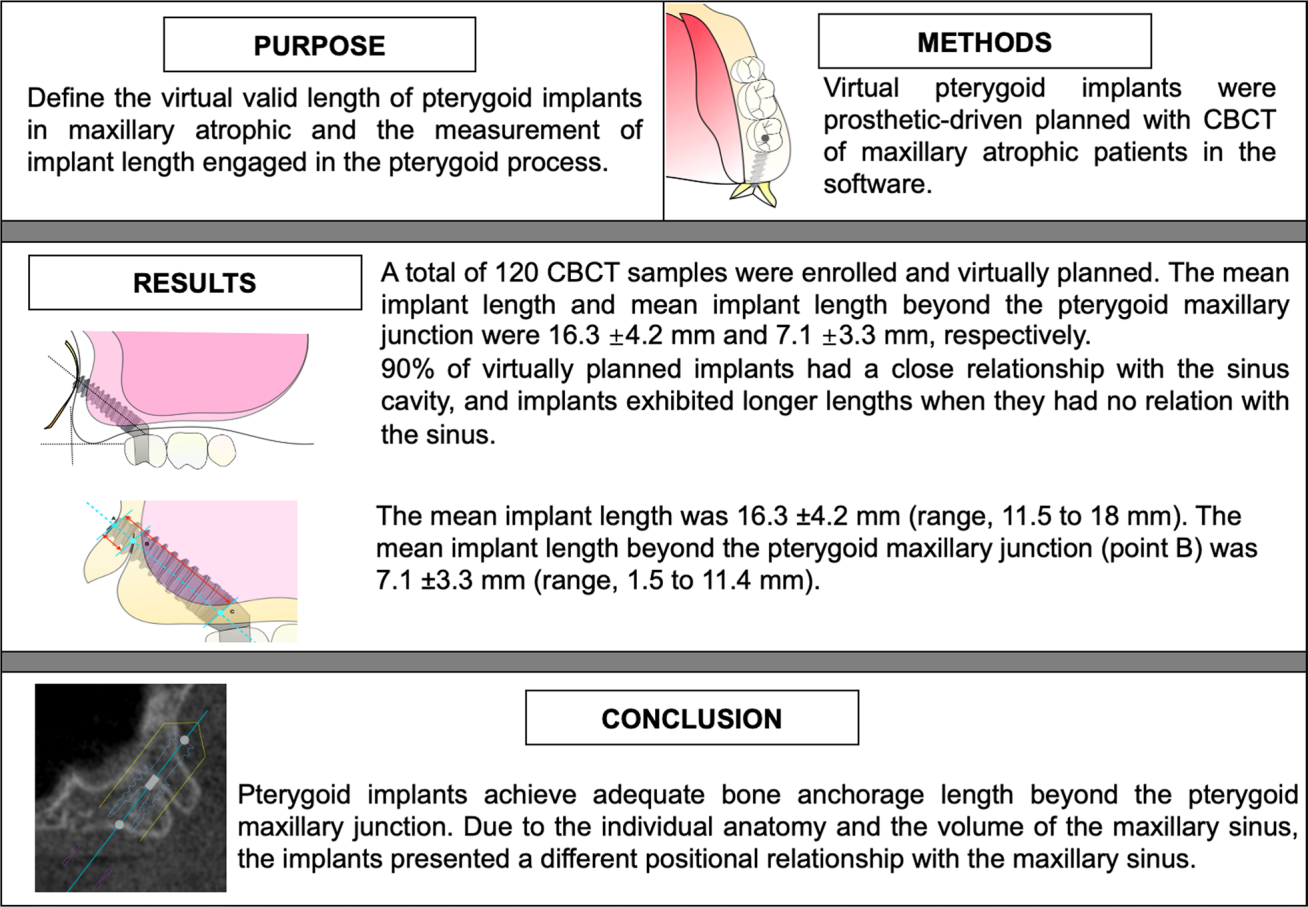

## Introduction

The pterygoid implant was proposed by Tuslane and Tessier in 1989 as a graftless solution to treat atrophic posterior maxilla [1, 2]. One recent review study with the enrollment of 634 patients and 1894 pterygoid implants revealed that pterygoid implants had a comparable high survival rate with conventional implants [3, 4].

Compared to sinus grafting, this approach is associated with several advantages, including reducing treatment duration and decreasing surgical morbidity and cost [5, 6]. From the prosthetic perspective of view, by maximizing the anterior–posterior spread and eliminating long distal cantilever, pterygoid implants provide beneficial biomechanical force during functional loading. In maxillary full edentulous situations with adequate primary stability and anchorage, pterygoid implants can help to make immediate loading feasible when combined with other axial conventional implants.

Although the concept and technique have been proposed for 30 years, due to the complex anatomical structure the implant passes through and its technique sensitivity (it is a semiblind procedure through 15 of 20 mm of bone), limited studies, mainly retrospective studies, and a limited number of implants inserted are available in the literature.

The implant passes through the maxillary tuberosity, pyramidal process of the palatine bone and pterygoid process of the sphenoid to pterygoid fossa. Some authors have described this anatomical complex as a “corridor”. In previous studies, to determine the ideal position of pterygoid implants, several measurements have been performed to determine the bone available for the ‘corridor’ and the safe and ideal position and angulation of pterygoid implants. Unlike the posterior maxillary alveolar ridge, the ‘corridor’, especially the pterygoid process, is not a tooth-dependent structure and is not related to tooth pathology or sinus pneumatization. Anatomical structure analysis from cadaver and radiography studies showed that dense bone at the juncture of the palatine and pterygoid processes is approximately 3–6 mm. With the implant tilted and placed with an angulation, it is easy to obtain 8–9 mm cortical bone anchorage for good primary stability and implant-to-bone direct contact [7–10].

From a clinical point of view, the ideal emergence of the fixture would be limited between the first and second molar regions but not distal to the second molar area and extended with angulation through the pterygoid process into the fossa. Excessive distal entrance will lead to difficulty in restoring, and oral hygiene maintenance after prosthesis delivery will be difficult. However, this point was not often a concern of many in vitro anatomical measurements and clinical studies. The anterior–posterior and implant sizes were always determined in the sinus cavity and posterior wall of the sinus. Nevertheless, very limited information available on this region especially focuses on prosthesis-driven implant placement. Regarding the issue of implant length, a variety of implant lengths between 13 and 20 mm were proposed by radiographic and anatomical studies. However, few studies have focused on valid implant lengths in the maxillary region and pterygoid process, and studies on the relationship between inserted implants and the sinus cavity are also lacking.

Regarding angulation, a range between 45° and 75° has been suggested after some investigations [8, 10]. Clinically, a distal 45° tilt is the most simplified angulation to judge and handle in implant surgery, and it is easier to obtain parallel implants with prefabricated abutments with axially placed implants. From a biomechanical point of view, in the finite element analysis of mesially tilted implants, a degree of 45 always seems to be a threshold of lower stress values in peri-implant loading strain. Thus, several authors advise that clinicians place implants with an inclination of 45° relative to the Frankfort plane.

The study based on cone beam computed tomography (CBCT) data aims to (1) define the virtual valid length of pterygoid implants in maxillary atrophic patients based on prosthetic data and (2) measure the implant length engaged in the pterygoid process according to the HU difference of the pterygoid maxillary junction.

## Materials and methods

### Patient selection

The study was approved by the ethical committee of Shanghai Ninth People’s Hospital, Shanghai Jiao Tong University, School of Medicine and was conducted according to the Helsinki Declaration.

Patients who had missing posterior maxillary teeth and presented atrophic posterior upper jaws were included. Patients had been edentulous for more than one year in the posterior maxillary region. CBCT scans using the i‐CAT 3D Imaging System (Imaging Sciences International, Hatfield, PA, USA) were performed using the following scanning parameters: 5 mA, 120 kV, voxel size of 0.4 mm, FOV of 25 cm (D) × 18 cm (H), and scan time of 16–20 s.

Patients who had residual bone height less than 4 mm due to sinus pneumatization or alveolar ridge resorption were enrolled. If the patient had bilateral posterior maxillary atrophy, the right side was chosen for evaluation to avoid individual error. Then, the CBCT data were exported as DICOM (Digital Imaging and Communications in Medicine) files and imported to planning software (Nobel Clinician, Nobel Biocare, Sweden) for anatomical structure measurement and pterygoid implant planning.

### Radiographic measurement

#### Virtual implant placement

Due to different alveolar ridge resorption patterns and the position of the head during CBCT scanning, the occlusal plane was not as stable as the Frankfort plane. Thus, the Frankfort plane (a line from the tragus of the ear through the infraorbital rim area) was used as a relatively horizontal line. Two independent investigators (YS and NW) performed the measurement, and interrater reliability was calculated.

**Fig. 1:**
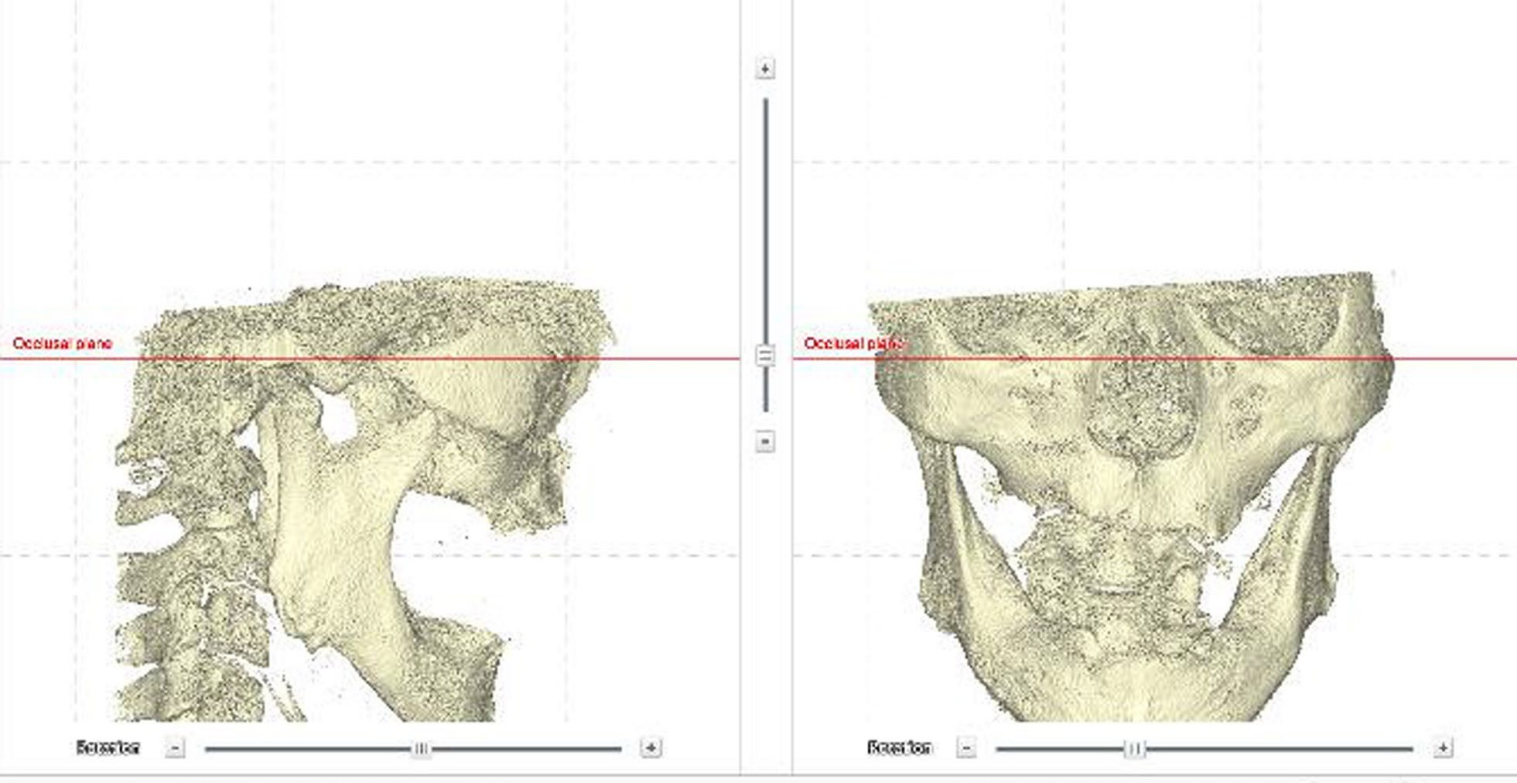
The Frankfort plane was first drawn in the software (Nobel Clinician)

**Fig. 2:**
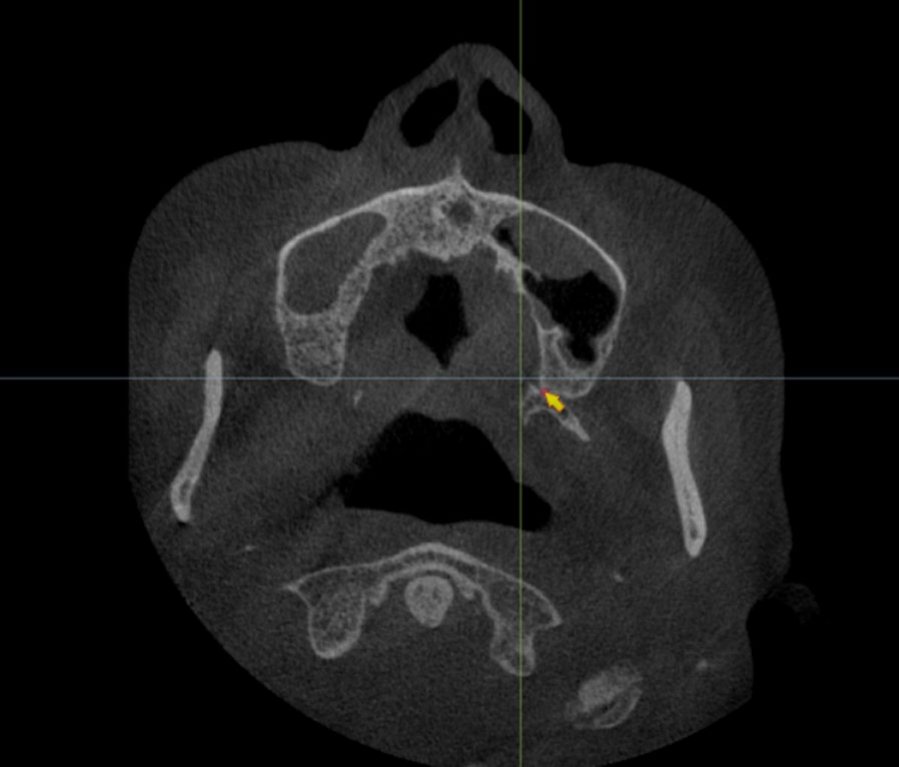
The lowest point of the pterygoid maxillary junction was first recognized between the maxillary tuberosity and pterygoid process from the coronal plane in the software

An implant that was 4.3 mm in diameter (Nobel Active, Nobel Biocare, Sweden) was virtually placed according to one unified plan. In the head position, the red line in the middle passes through the bilateral infraorbital points and the upper edge of the external auditory canal to determine the Frankfort plane (Fig. 1). The lowest point of the pterygoid maxillary junction was first recognized from the higher density according to HU value assessment between the maxillary tuberosity and pterygoid process from the coronal plane (Fig. 2).

**Fig. 3:**
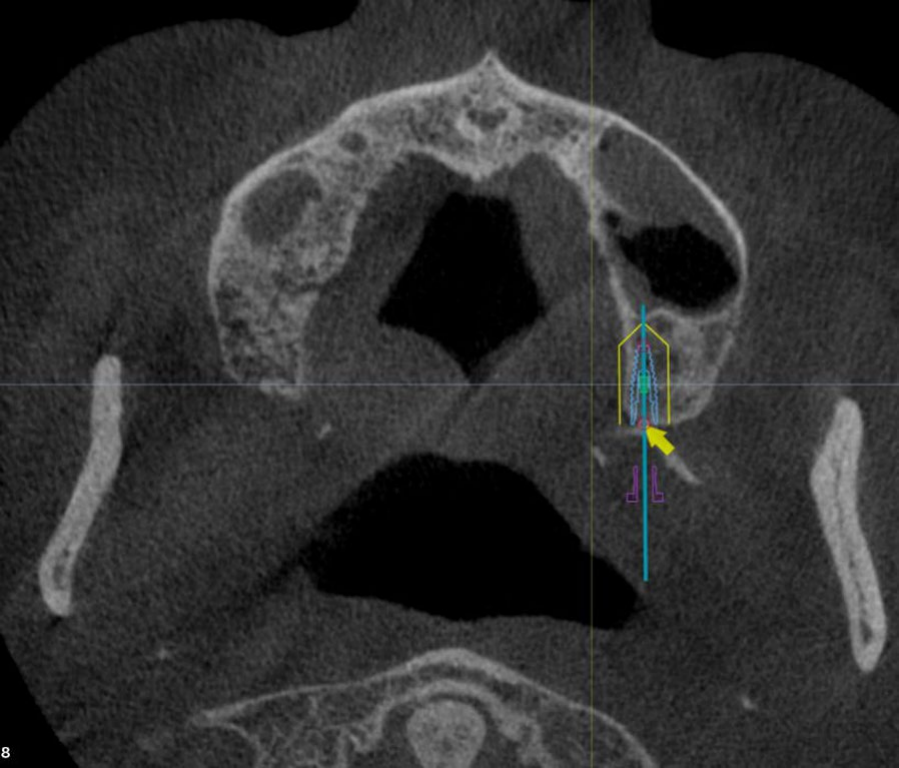
Create a 10 mm implant from the marked point forward and adjust the position of the implant so that it is parallel to the midline

**Fig. 4:**
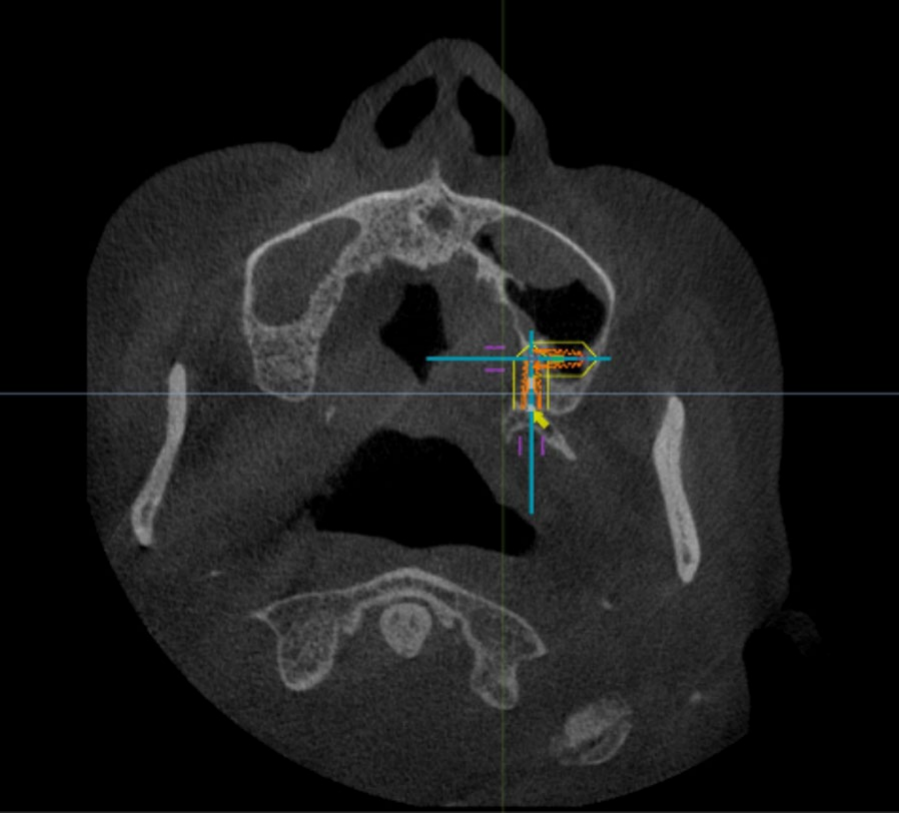
A 10 mm implant parallel to the coronal plane from the top of the previous implant was made

**Fig. 5:**
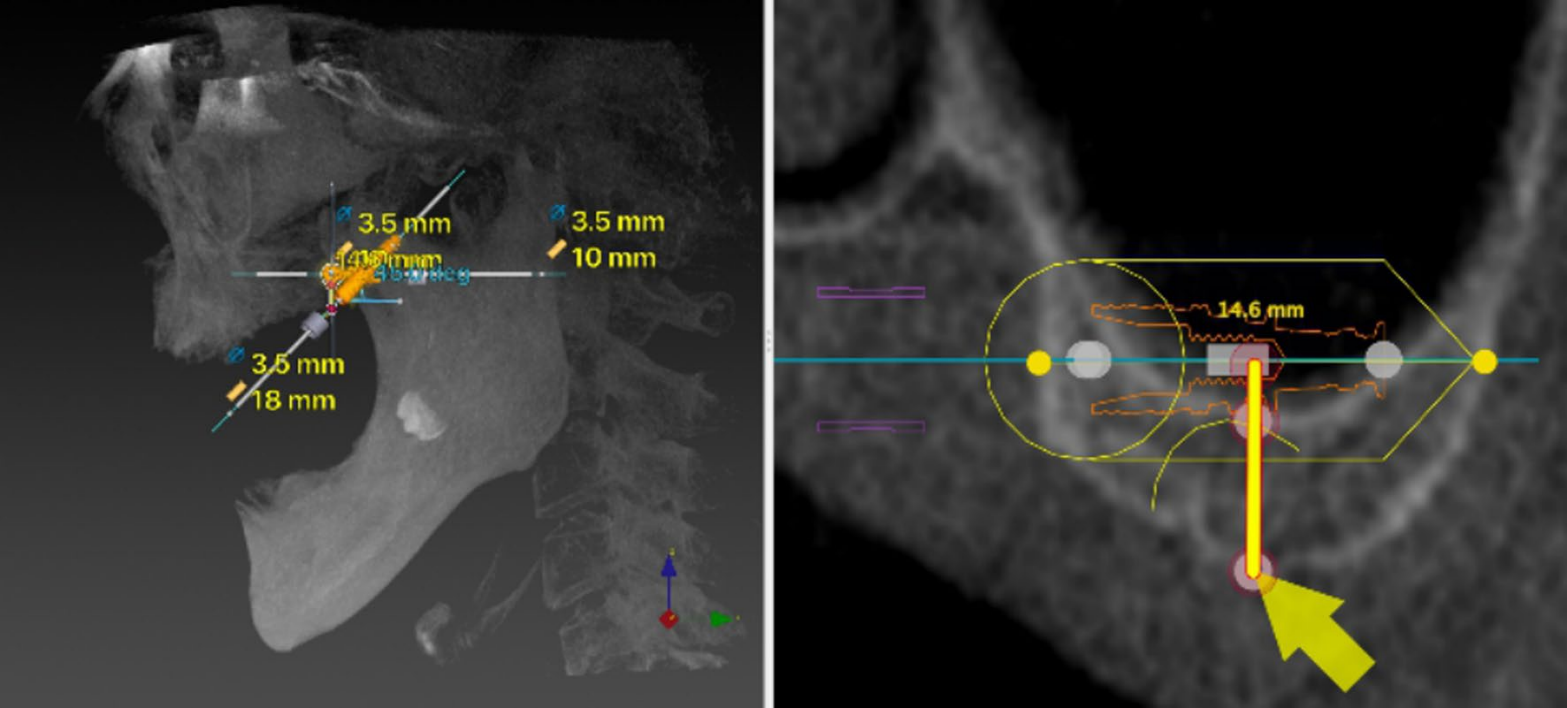
The interface of the software was adjusted to the coronal plane until the central part of the second virtually placed implant was observed. The midpoint of the alveolar ridge was confirmed as the entrance point of the pterygoid implant

**Fig. 6:**
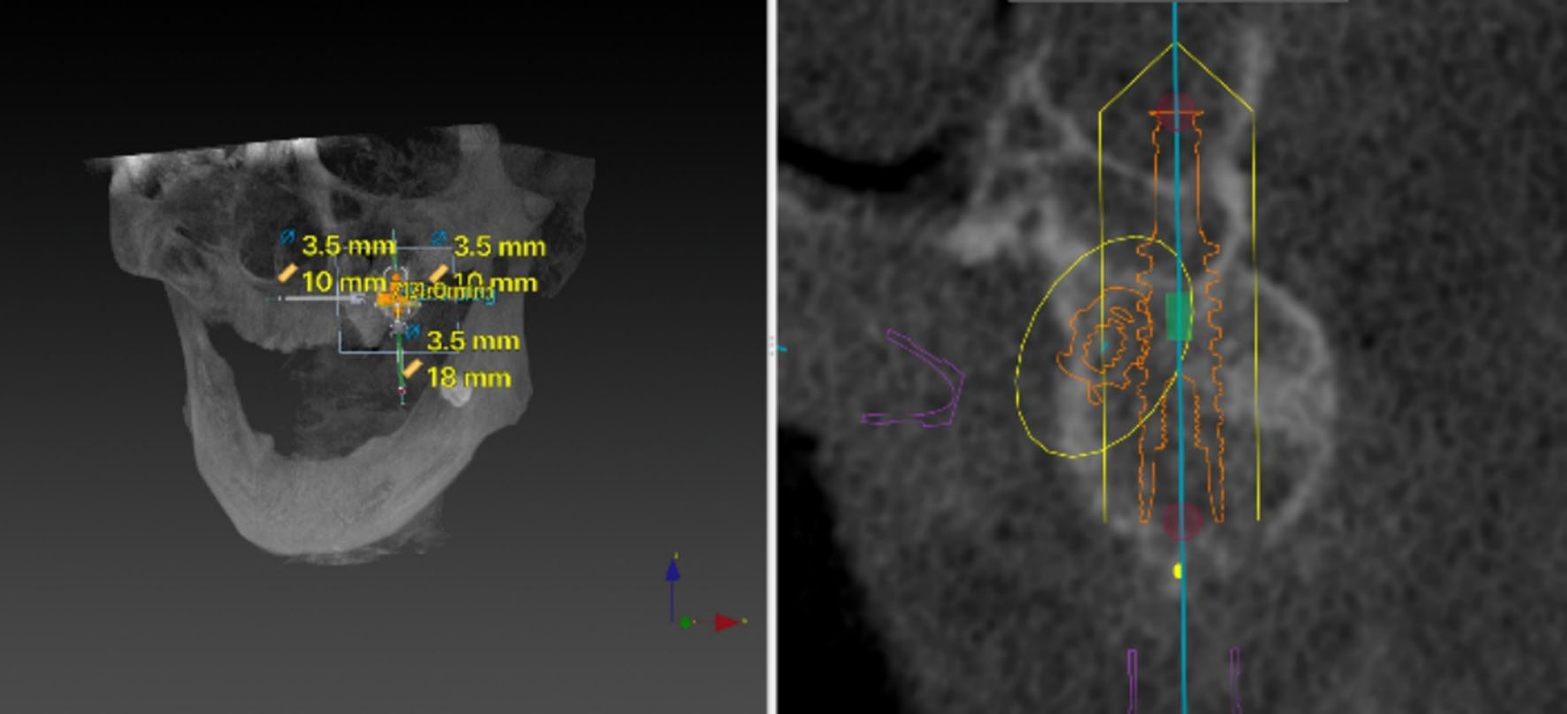
An 11.5–18 mm implant was virtually planned at an inclination of 45° relative to the Frankfort plane until reaching the pterygoid fossa

**Fig. 7:**
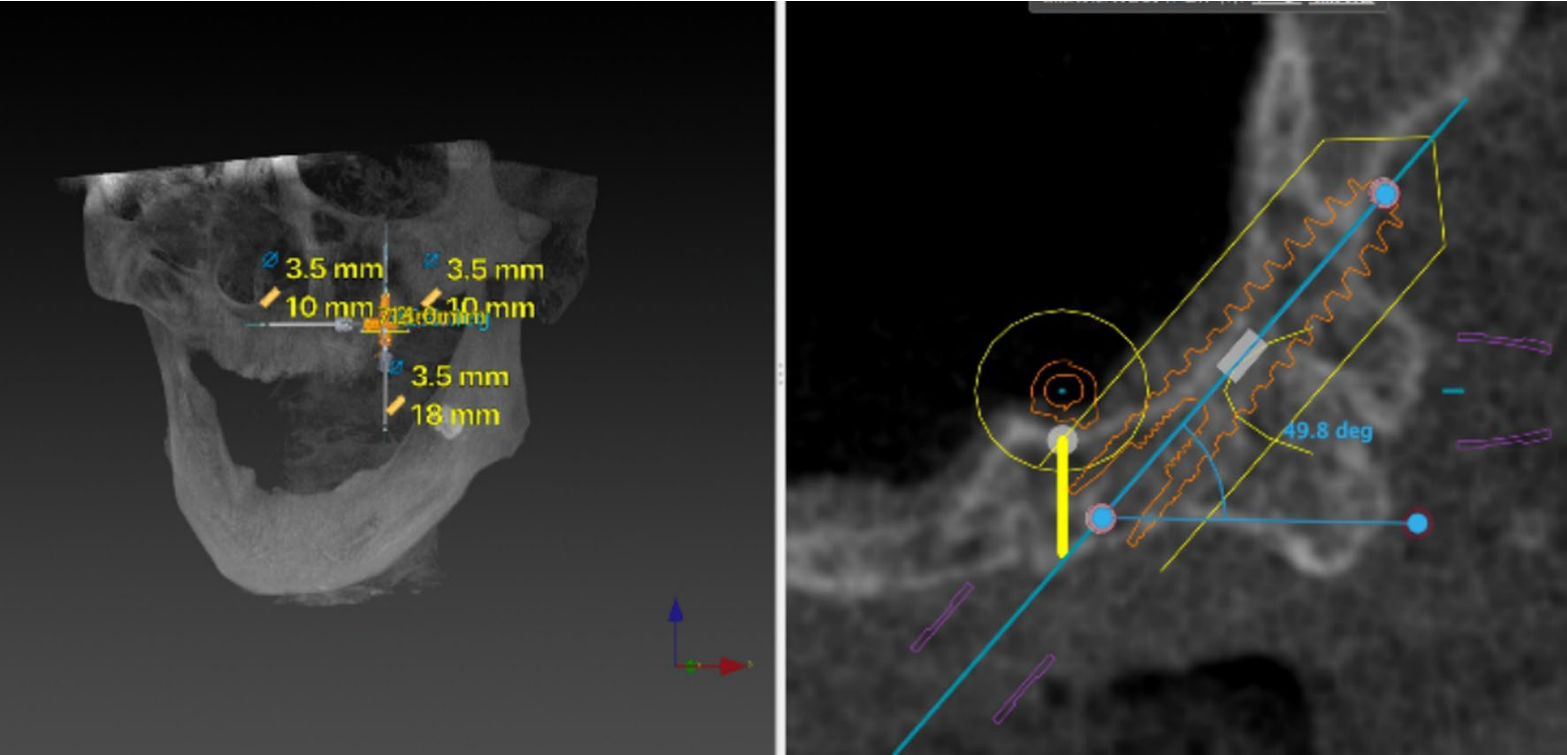
Maintain the inclination of the implant and adjust the implant apical exit point to penetrate the most concave cortical layer of the pterygoid process

Then, a virtual implant entrance point was set 10 mm away from the point parallel to the midline [11, 12]. Later, a 10 mm implant parallel to the coronal plane from the top of the previous implant was made (Figs. 3 and 4). The interface of the software was then adjusted to the coronal plane, and the entrance point at the midpoint of the alveolar ridge was confirmed (Fig. 5). An 11.5–18 mm implant was virtual planned at an inclination of 45° relative to the Frankfort plane until reaching the pterygoid fossa. The exit point was the most concave point where the implant penetrated the cortical layer of the pterygoid process (Figs. 6, 7).

### Parameters measurement

**Fig. 8:**
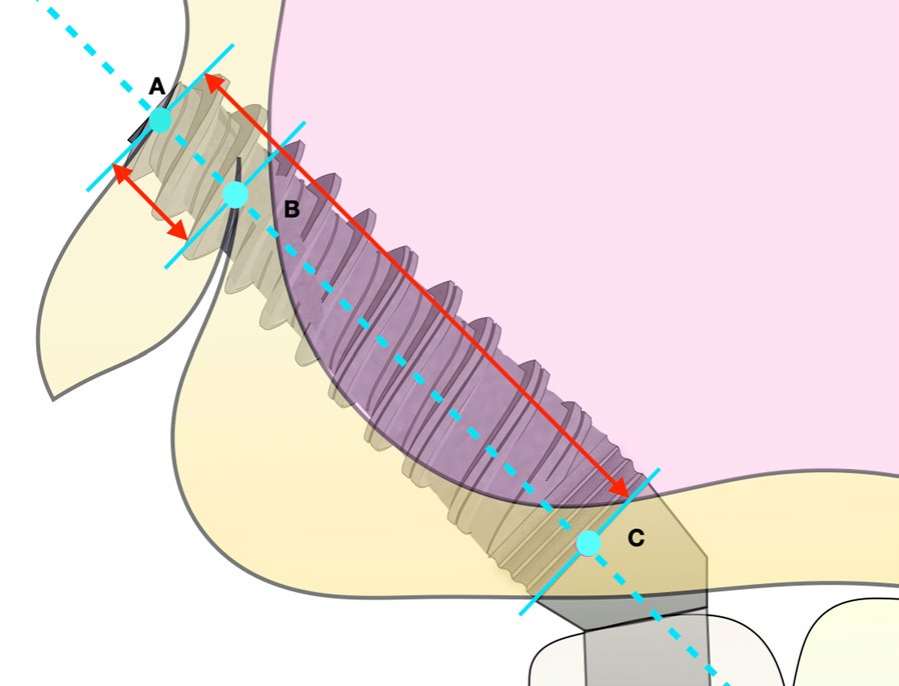
When the implant (point A–point C) beyond the pterygoid maxillary junction (point B) was considered that implant apex anchorage in the pterygoid process, and this part of implant length was measured (point A–point B)

The virtually placed implant length was recorded. In the 3D reconstructed module, the implant length in the sinus cavity was also recorded. The implant beyond the pterygoid maxillary junction was considered an implant in the pterygoid process, and the length of this implant was also measured (Fig. 8). In addition to the length of the implant measured in each anatomical structure, the buccal-palatal angulation of the implant in coronal sections was also calculated.

**Fig. 9:**
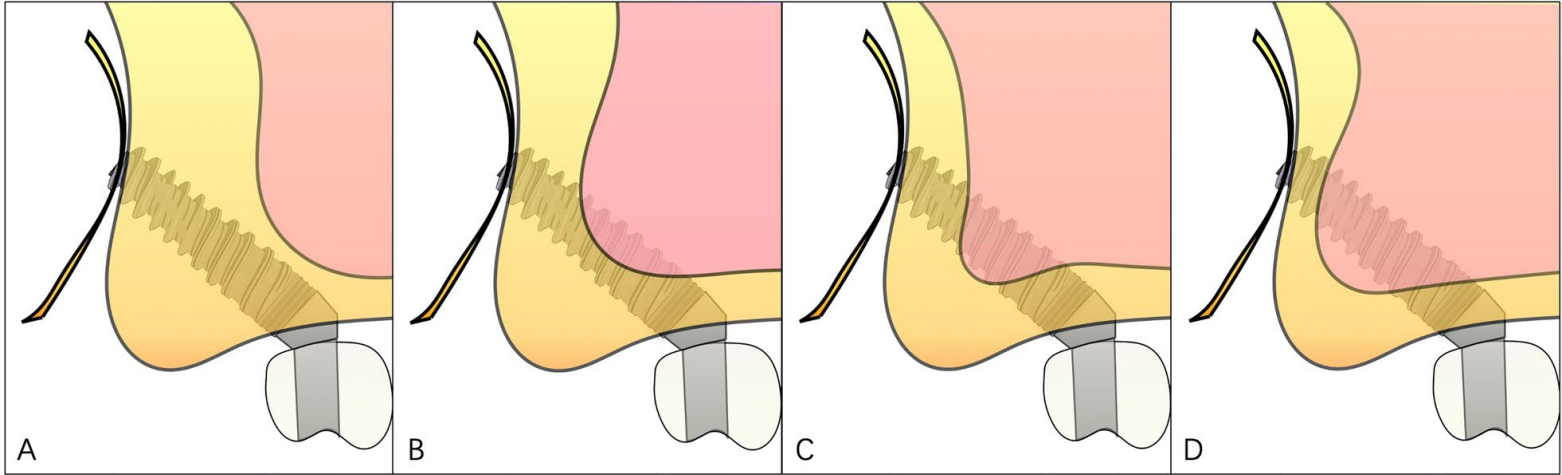
Four categories of relationship between the body of the implant and the sinus cavity. **A** No implant body in the sinus cavity. **B** One side of implant anchored in the posterior sinus wall and the other side of implant in the sinus cavity. **C** Less than 1/2 of the whole implant body in the sinus cavity. **D** Greater than 1/2 of the whole implant body in the sinus cavity

The relationship between the body of the implant and the sinus cavity was divided into 4 categories. A: No implant body in the sinus cavity; B: One side of implant anchored in the posterior sinus wall and the other side of implant in the sinus cavity; C: Less than 1/2 of the whole implant body in the sinus cavity; D: Greater than 1/2 of the whole implant body in the sinus cavity (Fig. 9).

### Statistical analysis

Statistical analysis was performed using SPSS software (SPSS, Inc., Chicago, IL, USA). Cronbach’s α coefficient (α) was obtained to verify interrater reliability. Descriptive statistics of the virtually planned implant values were calculated. The normality of the data was corroborated with the Kolmogorov–Smirnov test.

## Results

A total of 120 CBCT samples were enrolled and virtually planned. The mean age of the patients was 56.2 ± 13.2 years (range, 32–64 years old). The mean lost teeth in this group of patients was 7.8 (range, 3–14) in the maxillary arch, and the etiology of tooth loss was periodontitis.

The coefficient (α) was 0.86, showing good inter-rater reliability. According to the above-mentioned placement principle, 4 of 120 implants could not penetrate from the pterygoid fossa. The remaining 116 CBCT samples were enrolled and virtually planned.

The number and percentage of virtually planned implants with different lengths are listed in Table 1. The software provides virtually placed implants with fixed lengths. Among these lengths, 15 and 18 mm had the highest percentages. The mean implant length was 16.3 ± 4.2 mm (range, 11.5–18 mm). The mean implant length beyond the pterygoid maxillary junction was 7.1 ± 3.3 mm (range, 1.5–11.4 mm). The implant proportion distribution of different lengths beyond the pterygoid maxillary junction is shown in Table 2.

**Table 1:** The number and percentage of virtually planned implants with different lengths in 116 samples

	Implant length (mm)				
	11.5	13	15	18	Total
Number	3	9	44	60	116
%	2.6	7.7	37.9	51.7	100

**Table 2:** Implant length beyond the pterygoid maxillary junction

	Implant length beyond the pterygoid maxillary junction (mm)						
	0–2	2–4	4–6	6–8	8–10	10–12	Total
Number	2	14	45	47	7	1	116
%	1.7	12.1	38.8	40.5	6.0	0.9	100

Eleven of 116 implants (9.4%) showed no relationship to the sinus cavity. The implant passed the large maxillary tuberosity directly through the pterygoid process. In this category, the mean virtually planned implant length was 17.2 ± 2.4 mm (range, 13–18 mm), which was slightly longer than the mean values throughout the study. In categories B and C, the numbers of virtually planned implants were 12 (10.3%) and 65 (56.0%), respectively. The other 28 implants (24.1%), which belonged to category D, exhibited a closer relationship to the sinus cavity. Greater than half the length of the implant went into the maxillary sinus. The implant proportion distribution of different lengths in the maxillary sinus cavity is shown in Table 3.

**Table 3:** Implant length in maxillary sinus cavity

	Implant length in maxillary sinus cavity (mm)				
	0–5	5–8	8–12	12–16	Total
Number	19	48	33	16	116
%	16.4	41.4	28.4	13.8	100

The mean buccal-palatal angulation of the implant in coronal sections was 83.2 ± 3.02° (81.3–85.6°).

## Discussion

Posterior maxillary teeth loss due to periodontitis is a common clinical scenario. Compared to bone grafting, pterygoid maxillary implants provide an alternative solution in making the best use of residual bone of the maxillary-pterygoid complex. In contrast to the bone quality of the posterior maxilla, the implant engaged in the cortical layer of the pterygoid process makes it easy to obtain optimum primary stability [13–15]. Although this approach has been proposed for more than 30 years, its application is still limited. The anatomical complex of the maxillary-pterygoid complex constrains its clinical usage; moreover, clinicians are concerned about the entrance location of the implant for later maintenance.

To analyze the anatomical characteristics of this complex from a clinically feasible perspective, the present virtual study sought to provide information related to this complex from prosthetic-driven implant placement.

In previous anatomical measurements, the distance between the most concave point on the lateral surface of the pterygomaxillary junction and the greater palatine foramen was approximately 7 mm. With the horizontal and vertical absorption of alveolar bone, the distance between the aforementioned structures is reduced. The study set the entrance point of the virtual implant 10 mm away from the junction in the horizontal plane to mimic the entrance location of the second molar area and to proceed from the perspective of being more clinically feasible and maneuverable.

The inclination of pterygoid implants has been studied in previous radiographic and cadaver studies [16]. The range of 45–74° to the Frankfort plane in the anterior–posterior axis was proposed, and 45° was verified. Over a certain length with greater angles and more upright implants, the tip more easily reaches the pterygopalatine fossa, and the relationship with the maxillary artery will be closer. Undoubtedly, in clinical practice, implant placement is planned and driven by the individual anatomical character of each patient. However, compared to other angulations, tilted implants with 45° are easier to handle in clinical practice. Therefore, we set this criterion to virtually place the pterygoid maxillary implant.

In the assessment of buccal-palatal angulation, due to the fixed entrance and exit position of the virtually placed implant, it is easy to calculate the angulation in the software. A minor difference was detected between the present study and previous reports. In Rodriguez et al.’s study, an implant angulation of 81.09 ± 2.65° were found in the buccal-palatal axis [17]. To avoid the maxillary sinus cavity, every implant was placed according to individual anatomical structure in this study. Anatomically, the most concave cortical layer of the pterygoid process was in the palatal position of the alveolar ridge; when the implant entry moved more posteriorly, the angle of its buccal and palate reaching the most concave point became greater. In our study, the position of the implant entry point and the anterior–posterior inclination were determined, which explained the difference.

In the present study, the length of the implant beyond the pterygoid maxillary junction was the parameter of greatest concern. In some CBCT samples, the alveolar process of maxillary bone and pterygoid process of sphenoid bone are not completely fused; in this situation, the pterygoid maxillary junction is very easy to identify. Other samples showed a fused line of higher density compared to the surrounding structures. In contrast to large volume changes in the alveolar process and maxillary sinus cavity, the bone volume and density of the sphenoid pterygoid and palatine vertebrae are basically constant. These structures are precisely the most important structures that provide good stability of the implant during surgery.

Previous studies showed a mean bone column length (bone corridor) following the long axis of the implant of approximately 22 mm [1, 10, 18]. Some clinical studies established a minimal length of 13 mm for pterygoid implants. A deviation in implant length beyond the junction was detected in the study, and the range of 4–8 mm was the most common length.

We found that the limited anchorage of some implants in the pterygoid process is too short, usually when the sinus cavity is large. In addition, the maxillary tubercle is too small, and the posterior wall of the maxillary sinus is located behind the entrance of the pterygoid implant. When the implants had bone anchorage in the maxillary tuberosity, in category A, the mean virtually planned implant length was longer than the average length.

To establish a standardized entry point, the size of the maxillary tuberosity still affects the choice of implant length. Four patients could not complete the design because the vertical bone resorption of the alveolar ridge was too severe. After implantation at an oblique angle of 45°, the implant tip was at the root of the pterygoid process or close to the direction of the pterygopalatine fossa. This finding suggests that in clinical practice, when the alveolar bone is absorbed vertically instead of the lack of bone height caused by maxillary sinus pneumatization, oblique angle implantation is very risky. The slope should be increased. When the vertical defect of the alveolar ridge is not too serious, the inclination angle of the implant can be appropriately reduced, for example, between 45° and 70°.

## Conclusion

With the limitations of this study, from the prosthetic prioritized driven position, the virtual valid length of pterygoid implants in maxillary atrophic patients was determined. An implant with a mean length of 16 mm could be used in this region.

This area could be implanted with an average length of 16 mm implants. Approximately half of this length could cross the pterygoid maxillary junction using the pterygoid process for retention, whereas the other half of the length is located in the maxillary tuberosity area. Due to the individual anatomy and the volume of the maxillary sinus, the implants presented a different positional relationship with the maxillary sinus.

## Data Availability

Raw data were generated at shanghai 9th people hospital. Derived data supporting the findings of this study are available from the corresponding author WF and SCF on request.

## Abbreviations

CBCT: Cone beam computed tomography
DICOM: Digital imaging and communications in medicine

## References

[1] Tulasne JF, Worthington P, Brånemark PI (1992). Osseointegrated fxtures in the pterygoid region. Advanced osseointegration surgery, applications in the maxillofacial region.

[2] Balshi TJ, Lee HY, Hernandez RE (1995). The use of pterygomaxillary implants in the partially edentulous patient: a preliminary report. Int J Maxillofac Implants.

[3] Candel E, Penarrocha D, Penarrocha M (2012). Rehabilitation of the atrophic posterior maxilla with pterygoid implants: a review. J Oral Implantol.

[4] Balshi TJ, Wolfnger GJ, Balshi SF (1999). Analysis of 356 pterygomaxillary implants in edentulous arches for fixed prosthesis anchorage. Int J Oral Maxillofac Implants.

[5] Bidra AS, Huynh-Ba G (2011). Implants in the pterygoid region: a systematic review of the literature. Int J Oral Maxillofac Surg.

[6] Bahat O (1992). Osseointegrated implants in the maxillary tuberosity: report on 45 consecutive patients. Int J Oral Maxillofac Implants.

[7] Yamakura T, Abe S, Tamatsu Y, Rhee S, Hashimoto M, Ide Y (1998). Anatomical study of the maxillary tuberosity in Japanese men. Bull Tokyo Dent Coll.

[8] Rodríguez X, Méndez V, Vela X, Segalà M (2012). Modified surgical protocol for placing implants in the pterygomaxillary region: clinical and radiologic study of 454 implants. Int J Oral Maxillofac Implants.

[9] Salinas-Goodier C, Rojo R, Murillo-González J (2019). Juan Carlos Prados-Frutos: three-dimensional descriptive study of the pterygomaxillary region related to pterygoid implants: a retrospective study. Sci Rep.

[10] Grave SL (1994). The pterygoid plate implant: a solution for restoring posterior maxilla. Int J Periodont Rest Rent.

[11] Chrcanovic BR, Custódio ALN (2010). Anatomical variation in the position of the greater palatine foramen. J Oral Sci.

[12] Bahşi I, Orhan M, Kervancıoğlu P, Yalçın ED (2019). Morphometric evaluation and clinical implications of the greater palatine foramen, greater palatine canal and pterygopalatine fossa on CBCT images and review of literature. Surg Radiol Anat.

[13] Fernandez Valeron J, Fernandez Velazquez J (1997). Placement of screw-type implants in the pterygomaxillary-pyramidal region: surgical procedure and preliminary results. Int J Oral Maxillofac Implants.

[14] Valeron JF, Valeron PF (2007). Long-term results in placement of screw-type implants in the pterygomaxillary-pyramidal region. Int J Oral Maxillofac Implants.

[15] Franchina A, Stefanelli LV, Gorini S, Fedi S, Lizio G, Pellegrino G (2020). Digital approach for the rehabilitation of the edentulous maxilla with pterygoid and standard implants: the static and dynamic computer-aided protocols. Methods Protoc.

[16] Araujo RZ, Júnior JFS, Cardoso CL, Condezo AFB, Júnior RM, Curi MM (2019). Clinical outcomes of pterygoid implants: systematic review and meta analysis. J Cranio Maxillo Facial Surg.

[17] Rodríguez X, Rambla F, De Marcos L, Lopez VM, Vela X (2014). Jaime Jiménez Garcia: Anatomical study of the pterygomaxillary area for implant placement: cone beam computed tomographic scanning in 100 patients. Int J Oral Maxillofac Implants.

[18] Rodrıguez X, Lucas-Taule E, Elnayef B, Altuna P, Gargallo-Albiol J, Penarrocha MD, Hernandez-Alfaro F (2015). Anatomical and radiological approach to pterygoid implants: a cross sectional study of 202 cone beam computed tomography examinations. Int J Oral Maxillofac Surg.

